# Unexpected triggers for pheochromocytoma-induced recurrent heart failure

**DOI:** 10.1186/1755-7682-7-30

**Published:** 2014-06-21

**Authors:** Tiago Pereira-da-Silva, João Abreu, Ruben Ramos, Ana Galrinho, Philip Fortuna, Nuno Jalles Tavares, Rui Cruz Ferreira

**Affiliations:** 1 Department of Cardiology, Hospital de Santa Marta, Centro Hospitalar de Lisboa Central, Lisbon, Portugal; 2 Emergency and Intensive Care Department, Hospital de São José, Centro Hospitalar de Lisboa Central, Lisbon, Portugal; 3 MRI Center of Caselas, Lisbon, Portugal

**Keywords:** Body massage, Cardiogenic shock, Heart failure, Pheochromocytoma, Pregnancy

## Abstract

Pheochromocytoma crisis typically presents as paroxysmal episodes of headache, tachycardia, diaphoresis or hypertension. We describe an uncommon case of recurrent non-hypertensive heart failure with systolic dysfunction in a young female due to pheochromocytoma compression. It presented as acute pulmonary oedema while straining during pregnancy and later on as cardiogenic shock after a recreational body massage. Such crisis occurring during pregnancy is rare. Moreover, of the few reported cases of pheochromocytoma-induced cardiogenic shock, recreational body massage has not yet been reported as a trigger for this condition.

## Introduction

Pheochromocytoma is an endocrine tumour that typically presents as paroxysmal episodes of headache, tachycardia, diaphoresis or hypertension due to increased catecholamine release, although many patients are asymptomatic and the condition may be incidentally diagnosed on imaging modalities [1,2]. Non-hypertensive heart failure presenting as acute pulmonary oedema or cardiogenic shock due to pheochromocytoma is uncommon [3,4]. We report an atypical case of pheochromocytoma causing recurrent non-hypertensive heart failure with systolic dysfunction, which is unique considering the triggers. A review of the reported triggers for pheochromocytoma-induced cardiogenic shock is included.

## Case report

A 41-year-old white female with no relevant past medical history became pregnant at 35 years old, which was the only pregnancy to date. At 25 weeks of gestation, while straining by lifting weights, she suddenly developed acute pulmonary oedema with mild hypotension (81/42 mmHg). The ECG showed sinus tachycardia, associated to mild troponin I elevation (0.97 ng/mL; 0–0.06), high brain natriuretic peptide levels (750 pg/mL; 0–100) and a left ventricular ejection fraction (LVEF) of 38%, with global hypokinesia on transthoracic echocardiogram (TTE). No other symptoms or signs including fever, or drug abuse were reported. Preeclampsia was ruled out as the patient was mild hypotensive and did not have proteinuria or peripheral edema. No other laboratory abnormalities were found, particularly regarding the inflammatory makers and liver enzymes. Viral screening for enterovirus including echo and coxsackie, adenovirus, parvovirus, cytomegalovirus and Ebstein-Barr virus was negative, and autoimmune screening including antinuclear antibodies, anticardiolipin antibodies, anti-b2GP1, antineutrophil cytoplasmic antibodies, C3 and C4 was unremarkable. Computed tomography angiography excluded pulmonary embolism. The patient improved in 48 hours on intravenous furosemide with complete LVEF recovery. No further complications occurred, including at the delivery at 37 weeks, which excluded peripartum cardiomyopathy.

**Figure 1:**
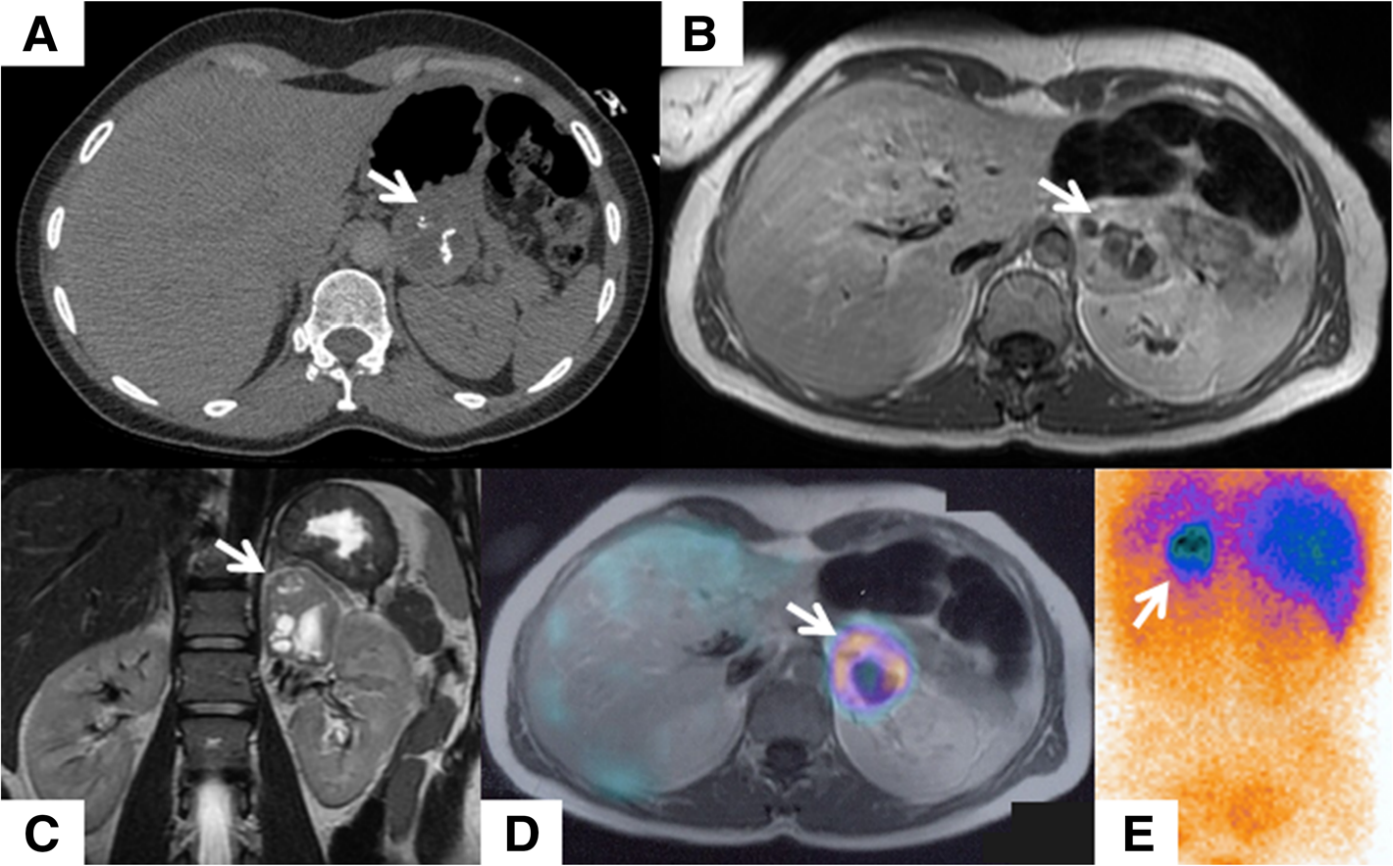
**Pheochromocytoma imaging features.** Pheochromocytoma mass (arrows) with heterogeneous appearance in computed tomography **(A)**, revealing hypointense signal on T1-weighted **(B)** and hyperintense signal on T2-weighted **(C)** sequences of magnetic resonance imaging, and high uptake on ^123^I-mIBG scintigraphy **(D,E)**.

**Figure 2:**
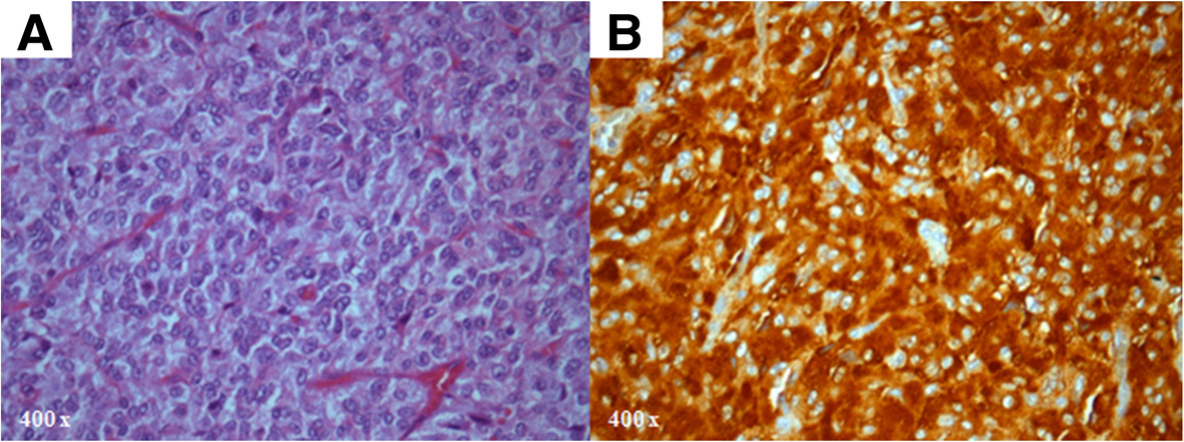
**Histologic analysis.** Nested arrangement of cells (Zellballen) with large cytoplasm and prominent nucleolus (HE, 400×) **(A)**, and immunohistochemical staining for synaptophysin (400×) **(B)**, confirming the diagnosis of pheochromocytoma.

The patient remained asymptomatic until 41 years of age when, during a recreational body massage with compression of the left flank, she developed cardiogenic shock with hypotension (65/35 mmHg) and oligoanuria. She was stabilized with dopamine. The TTE revealed severe systolic dysfunction with a LVEF of 20% with global hypokinesia and moderate functional mitral regurgitation (see videos, Additional files 1 and 2). Aetiological investigation similar to that undertaken in the first episode revealed identical findings. After ruling out pregnancy, coronary angiography excluded coronary lesions. A complete clinical and echocardiographic recovery occurred in 36 hours (see video, Additional file 3). The cardiac magnetic resonance imaging (MRI) performed three days after recovery was unremarkable, including for the absence of left ventricle wall oedema or lade gadolinium enhancement. After reviewing the computed tomography images, performed on admission for pulmonary embolism exclusion, an heterogeneous 5 × 5 cm mass was disclosed in continuity with the left adrenal gland. MRI findings were suggestive of pheochromocytoma and the mass showed high uptake on ^123^I-mIBG scintigraphy (Figure 1). Serum epinephrine (185 pg/mL; 0–125) and urinary epinephrine (159 μg/day; 0–22), metanephrines (6116 μg/day; 0–302) and vanilmandelic acid (13.6 mg/day; 0–6.7) levels were elevated, after one week of dopamine washout. The mass was excised and histological analysis confirmed the diagnosis of pheochromocytoma (Figure 2). At six months of follow-up there was no recurrence of heart failure.

## Discussion

This case of recurrent heart failure is a form of stress induced cardiomyopathy caused by catecholamine release due to pheochromocytoma. Straining in the presence of an enlarged uterus and later on the abdominal massage, both causing tumour compression, were the identified triggers. Spontaneous pheochromocytoma crises cannot be excluded. However, the strong temporal association between the reported triggers and heart failure onset as well as the anatomical location of the tumour as easily compressible make the association between the triggers and pheochromocytoma crises very likely.

Pheochromocytoma was not suspected in the first episode. It was not elicited in the first computed tomography angiography as its spatial window acquisition was limited as much as possible and an abdominal shield was used for protecting the pregnant abdomen, hampering the visualization of upper abdomen. In fact, patients may have a pheochromocytoma and be asymptomatic for long periods and up to 10% will not have symptoms at all, thus the tumour may be present for a long period before it is diagnosed [1,2]. It is likely that the tumour was already present during pregnancy considering its usual natural history. In addition, the presentation of the crisis during pregnancy was similar to the second episode in which pheochromocytoma was identified as the cause for heart failure, also supporting that pheochromocytoma was the culprit in the first episode.

Pheochromocytoma crisis during pregnancy is rare and it is usually associated with hypertension, although non-hypertensive acute pulmonary oedema and cardiogenic shock have been described [5-8]. The crisis mainly occurs close to delivery or following exposure to pharmacologic agents, which was not the case [7-9].

Regarding pheochromocytoma-induced cardiogenic shock, few cases have been described [1,3]. In addition to the rare cases associated with pregnancy, the reported triggers include nonadrenal abdominal and nonabdominal surgical procedures, infection such as pneumonia and pyelonephritis, spontaneous acute haemorrhagic necrosis of the pheochromocytoma, perforation of the colon, physical exercise and pharmacologic agents such as metoclopramide, steroids, dobutamine use including for stress echocardiography, betablockers including labetalol, propranolol and penbutolol, phentolamine, imipramine, prochlorperazine, phenothiazine and a combination of ergotamine, caffeine and nimesulide [3,9-21]. Two cases of cardiogenic shock due to pheochromocytoma following blunt trauma have been described, one of them in association to pheochromocytoma haemorrhage [22,23]. To our knowledge, a recreational body massage has not yet been reported as a trigger for pheochromocytoma-induced cardiogenic shock.

In the particular case of diagnosed pheochromocytoma, discouraging from abdominal recreational massage should be considered while waiting for definite surgical therapy.

## Conclusion

The patient had an atypical presentation of pheochromocytoma occurring with recurrent non-hypertensive heart failure with systolic dysfunction. Recreational body massage should be considered a trigger for this condition.

## Consent

Written informed consent was obtained from the patient for publication of this case report and any accompanying images. A copy of the written consent is available for review by the Editor-in-Chief of this journal.

## Abbreviations

CD: Colour doppler; LV: Left ventricle; LVEF: Left ventricular ejection fraction; MRI: Magnetic resonance imaging; PLAX: Parasternal long axis; TTE: Transthoracic echocardiogram.

## Competing interests

The authors declare that they have no competing interests.

## Authors’ contributions

TPS has participated in data collection, conception and design of the paper and drafting the manuscript. JA and RR have made substantial contributions to the conception and design of the paper and have been involved in drafting the manuscript. AG, PF and NJT have made substantial contributions in data collection and in revising the manuscript for important intellectual content. RCF has critically revised the manuscript for important intellectual content and gave final approval of the version to be submitted. All authors read and approved the final manuscript.

## Supplementary Material

Additional file 1 **Video A.** Transthoracic echocardiogram (TTE) on admission, parasternal long axis (PLAX) view with colour Doppler (CD): severely depressed left ventricle (LV) systolic function with global hypokinesia and functional mitral regurgitation. Click here for file

Additional file 2 **Video B.** TTE on admission, 4-chamber view: severely depressed LV systolic function with global hypokinesia. Click here for file

Additional file 3 **Video C.** TTE 36 hours after admission, PLAX view with CD: complete recovery of LV systolic function and of mitral regurgitation. Click here for file
